# Interaction of Biomechanical, Anthropometric, and Demographic Factors Associated with Patellofemoral Pain in Rearfoot Strike Runners: A Classification and Regression Tree Approach

**DOI:** 10.1186/s40798-023-00671-8

**Published:** 2024-01-08

**Authors:** José Roberto de Souza Júnior, Logan Walter Gaudette, Caleb D. Johnson, João Paulo Chieregato Matheus, Thiago Vilela Lemos, Irene S. Davis, Adam S. Tenforde

**Affiliations:** 1 Department of Physical Medicine and Rehabilitation, Spaulding Rehabilitation Hospital/Harvard Medical School, Boston, MA USA; 2 Graduate Program of Sciences and Technologies in Health, University of Brasília, Brasília, DF Brazil; 3 United States Army Research Institute for Environmental Medicine, Natick, MA USA; 4 Department of Physical Therapy, State University of Goiás, Goiânia, GO Brazil; 5 School of Physical Therapy and Rehabilitation Sciences, Morsani College of Medicine, University of South Florida, Tampa, FL USA; 6 Spaulding National Running Center, 1575 Cambridge St, Cambridge, MA 02138 USA

**Keywords:** Etiology, Biomechanics, Running injuries, Statistical approach

## Abstract

**Background:**

Patellofemoral pain (PFP) is among the most common injuries in runners. While multiple risk factors for patellofemoral pain have been investigated, the interactions of variables contributing to this condition have not been explored. This study aimed to classify runners with patellofemoral pain using a combination of factors including biomechanical, anthropometric, and demographic factors through a Classification and Regression Tree analysis.

**Results:**

Thirty-eight runners with PFP and 38 healthy controls (CON) were selected with mean (standard deviation) age 33 (16) years old and body mass index 22.3 (2.6) kg/m^2^. Each ran at self-selected speed, but no between-group difference was identified (PFP = 2.54 (0.2) m/s x CON = 2.55 (0.1) m/s, *P* = .660). Runners with patellofemoral pain had different patterns of interactions involving braking ground reaction force impulse, contact time, vertical average loading rate, and age. The classification and regression tree model classified 84.2% of runners with patellofemoral pain, and 78.9% of healthy controls. The prevalence ratios ranged from 0.06 (95% confidence interval: 0.02–0.23) to 9.86 (95% confidence interval: 1.16–83.34). The strongest model identified runners with patellofemoral pain as having higher braking ground reaction force impulse, lower contact times, higher vertical average loading rate, and older age. The receiver operating characteristic curve demonstrated high accuracy at 0.83 (95% confidence interval: 0.74–0.93; standard error: 0.04; *P* < .001).

**Conclusions:**

The classification and regression tree model identified an influence of multiple factors associated with patellofemoral pain in runners. Future studies may clarify whether addressing modifiable biomechanical factors may address this form of injury.

## Key Points

- This study highlights interactions between multiple biomechanical factors associated with patellofemoral pain.
- Interactions between braking ground reaction force impulse, contact time, vertical average loading rate, and age correctly identified runners with patellofemoral pain and controls.
- Step rate, sex, and body mass index were not predictors of patellofemoral pain in runners when a classification and regression tree analysis was used

## Background

Running is a popular activity that has clear cardiovascular and other health benefits [1]. However, participation in running has an annual rate of injury estimated at 19.4% to 79.3% [2], particularly in novice runners [3, 4]. The most common location of injury is the knee, and a strong risk factor for sustaining a knee injury is history of a previous injury in the past year [2, 5].

Patellofemoral Pain (PFP) is one of the most common types of knee injury in runners. It is defined as pain around or behind the patella during functional activities such as running, squatting, climbing and descending steps [6–8]. While conservative treatment can be effective, Collins and colleagues [9] found nearly half of patients had incomplete recovery at one year and Lankhorst and colleagues [10] identified over half of all patients may have incomplete recovery in 5–8 years.

Given that PFP is both common and challenging to treat, it is important to identify factors that may be related to PFP in runners [11–13]. Running biomechanical studies have added value in determining key kinetic and kinematic variables for a running-related injury (RRI) including PFP. Ground reaction force (GRF) variables associated with the impact phase of running, such as vertical loading rates have been associated with RRIs in general [14, 15] and specific to PFP [15]. However, other studies have found the opposite [16–18], suggesting the value of further study of these variables. In the anteroposterior direction, reduced braking ground reaction force impulse (BGRFI) has been noted in runners with PFP [19]. Additionally, greater contact time has been found in these runners [19]. Also, while step rate has not been associated with PFP [20], increases in step rate of 7.5–10% has been shown to reduce symptoms in those with PFP [21–23].

Other factors may also contribute to PFP. For example, while one study found no association of sex and PFP [13], two separate investigations have reported that females have approximately 2 times the incidence of PFP compared to their male counterparts [24, 25]. While higher body mass index (BMI) and age have not been found to be related to PFP [12, 26], further study is needed to determine whether they might interact with other factors to increase the risk of PFP.

Prior studies have explored risk factors for PFP; however, the association of different biomechanical, anthropometric, and demographic factors has not been consistent. These studies have been limited to addressing the bivariate associations between risk factors and PFP. The assumption of non-linearity must be considered in sports injuries [27]. It may be helpful to search for the interactions between risk factors to guide understanding of this condition [27, 28]. Regression models and Classification and Regression Trees (CART) are statistical approaches that can be used to explore risk factors for PFP. Regression models determine the average effect of an independent variable on an outcome while CART identifies subgroups of a population with common aspects that influence an outcome [29].

Complex problems, such as PFP, must be analyzed through the detection of interactions and not through the addition of factors. The main advantage of the CART analysis is that the assumption of linearity is not assumed and interactions between risk factors are identified by the development of clinical prediction rules. As a result, CART analysis may be better than regression models to identify profiles of runners that share common characteristics that are associated with PFP. Besides the identification of subgroups, CART produces a visual multilevel tree that is easy to interpret even when more than three variables are included in the model [29].

Previous studies using the CART approach have been able to identify runners with Achilles tendinopathy [30] and soccer players who sustained a re-injury of the anterior cruciate ligament [31]. The objective of this study was to identify different patterns of interaction among biomechanical, anthropometric, and demographic factors associated with PFP in runners using a CART analysis. We hypothesized that interactions involving various aspects of the GRFs and spatiotemporal variables, along with sex, age, body mass index, would correctly identify runners with and without PFP.

## Methods

### Study Design

This cross-sectional study followed the recommendations of STROBE (Strengthening the Reporting of Observational Studies in Epidemiology). Patients of the Spaulding National Running Center (SNRC) who were being treated for the primary condition of PFP were identified from clinic records and included as the injured cohort. A matched cohort of healthy runners were identified from a separate study.

### Setting

The SNRC was founded in 2012 and it is an ambulatory outpatient clinic that specializes in the diagnosis, treatment, and prevention of RRIs including PFP. A standard biomechanical evaluation is performed to guide physical therapy exercises and gait retraining, as needed. Participants were self-referred or referred from their sports physicians, orthopedic surgeons, and family physicians. Data were collected from September 2012 to July 2022 using standard procedures of collecting relevant history and gait assessment. Clinical charts were reviewed from August 2022 to October 2022.

### Participants

The injured group included runners with PFP who ran with a rearfoot strike pattern and were older than 18 years of age. Chart review of key aspects of history and examination findings were used to confirm the PFP diagnosis using the following criteria: pain around or behind the patella during running and at least one other task that engages the patellofemoral joint, including squatting, climbing, and descending steps, kneeling, or extending the knee with resistance [6]. Runners were excluded who presented with concomitant primary diagnoses, with underlying neurological/neuromuscular disorders, history of surgery in the lower extremity within six months of evaluation, or history of knee surgery at any time. Runners could have unilateral or bilateral symptoms, and the knee with highest pain intensity was used for analysis.

Healthy runners were identified from a study on the effects of footwear and foot strike pattern on running biomechanics. Participants were selected who were rearfoot strike runners with no RRIs 6 months prior the evaluation [30], no neurological/neuromuscular disorders, no history of surgery in the lower limbs in the 6 months prior to the evaluation, and no history of PFP. An equal number of healthy controls to injured runners were selected. Running speed is a potential confounding factor for biomechanical variables, so healthy participants were matched to the PFP group according to speed. The control runners came from a normative database of healthy runners. As speed has a significant impact on ground reaction forces, subjects were matched for speed. Also, participants had to run at least 10 miles/week to be included.

As the data were collected on injured runners to guide clinical treatment, the Institutional Review Board approved this protocol with waiver of informed consent (Protocol 2017P000481). Written informed consent was obtained from each healthy participant prior to participation in a study designed to develop a normative database of healthy runners (2012P002373).

### Data Collection

All participants (PFP and healthy control group (CON)) completed the same gait analysis on an instrumented treadmill embedded with two force plates (AMTI, Watertown, MA, USA; sampling rate = 1500 Hz). Sagittal plane high-speed video (125 fps) was used to determine foot strike pattern. Runners were classified as rearfoot strikers if they landed on the heel first [32]. A short warm-up (slow run of 2 to 3 min) was provided followed by instructions for each runner to increase speed to reach a self-selected running speed, described as a comfortable training pace for an easy training run [15, 33]. After reaching the self-selected speed, 10 consecutive foot strikes were collected for analysis. Runners with PFP ran in their own shoes. The healthy runners were part of a larger study that required lab-issued shoes to accurately measure foot kinematics. However, they were provided a type of shoe that matched their habitual footwear.

### Variables

The dependent variable was the presence of PFP. The independent variables included age, sex (male x female), BMI, vertical average loading rate (VALR), vertical instantaneous loading rate (VILR), braking ground reaction force impulse, contact time, and step rate.

### Biomechanical Data Processing

Ground reaction force data were filtered at a cut-off frequency of 50 Hz using a low pass, fourth-order Butterworth filter. A custom program written in MATLAB (MathWorks, Natick, Massachusetts USA) was used to process data as reported previously [15, 32, 33]. The point of interest (POI) was defined as the first point above 75% of a subject’s body weight (BW) with a vertical GRF slope less than 15 BW/s. The VALR (BW/s) region was defined as the largest region between 20 and 80% of the force at the POI with a continuous slope above 15 BW/s. The VILR (BW/s) was defined as the peak vertical load rate between any two successive points from 20 to 100% of the force at the POI. BGRFI (BW*s) was determined as the time integral of the anteroposterior GRF over stance [34]. Contact time (s) was considered as the time during which a vertical force greater than 10 N was applied to the force plate. Cadence was calculated in steps per minute.

### Sample Size/Bias

No a priori sample size calculation was performed as the sample was one of convenience. Measures to avoid potential sources of bias are described below: (I) rigorous criteria were chosen to classify patients in the patellofemoral pain group [6] and control group; (II) variables were chosen to be those that were related with PFP [13, 15–19, 24–26] or changes in symptoms in PFP runners [21–23]; (III) the matching process was done before gathering biomechanical variables to eliminate selection bias.

### Statistical Analysis

IBM SPSS (Statistical Package for Social Sciences, v.25) and Open-Epi were used to perform the statistical analysis. Normality was assessed using Shapiro–Wilk test and histograms. Parametric data were described as mean and standard deviation (SD) and non-parametric data as median and interquartile range (IQR). Categorical data were described as frequencies and percentages. A correlation matrix was performed within biomechanical variables to detect collinearity. In the case of collinearity (r > 0.71), only the most important variable was kept in the model. This was done to prevent splits that could overlap each other. A significance level of *P* < 0.05 was used.

The interactions between independent variables were assessed using a CART analysis. The predictors and their respective cut-off values that best classify the participants regarding the presence and absence of PFP were selected through CART. The predictors were selected based on the strength of association with the dependent variable (presence of PFP). The CART model begins with the total sample (node 0), and it is divided into 2 groups (sub-nodes) according to the best predictor and specific cut-off values. This process is applied recursively until the subgroups reach a minimum size or no improvement can be done (terminal node) [29, 35]. In the end, a tree representing the non-linear relationship among predictors that best classify the participants with and without patellofemoral pain is obtained. The criteria to produce the partitions were a minimum of 14 participants in each node to make a division and a minimum of 7 participants to generate a node [36]. Gini index of 0.0001 was used to maximize the node’s homogeneity and a tenfold cross-validation to avoid overfitting. Finally, a receiver operating characteristic (ROC) curve was created to verify the accuracy of the model and prevalence ratios (PR) with 95% confidence intervals (CI) were calculated for each terminal node to investigate the strength of associations.

## Results

**Fig. 1:**
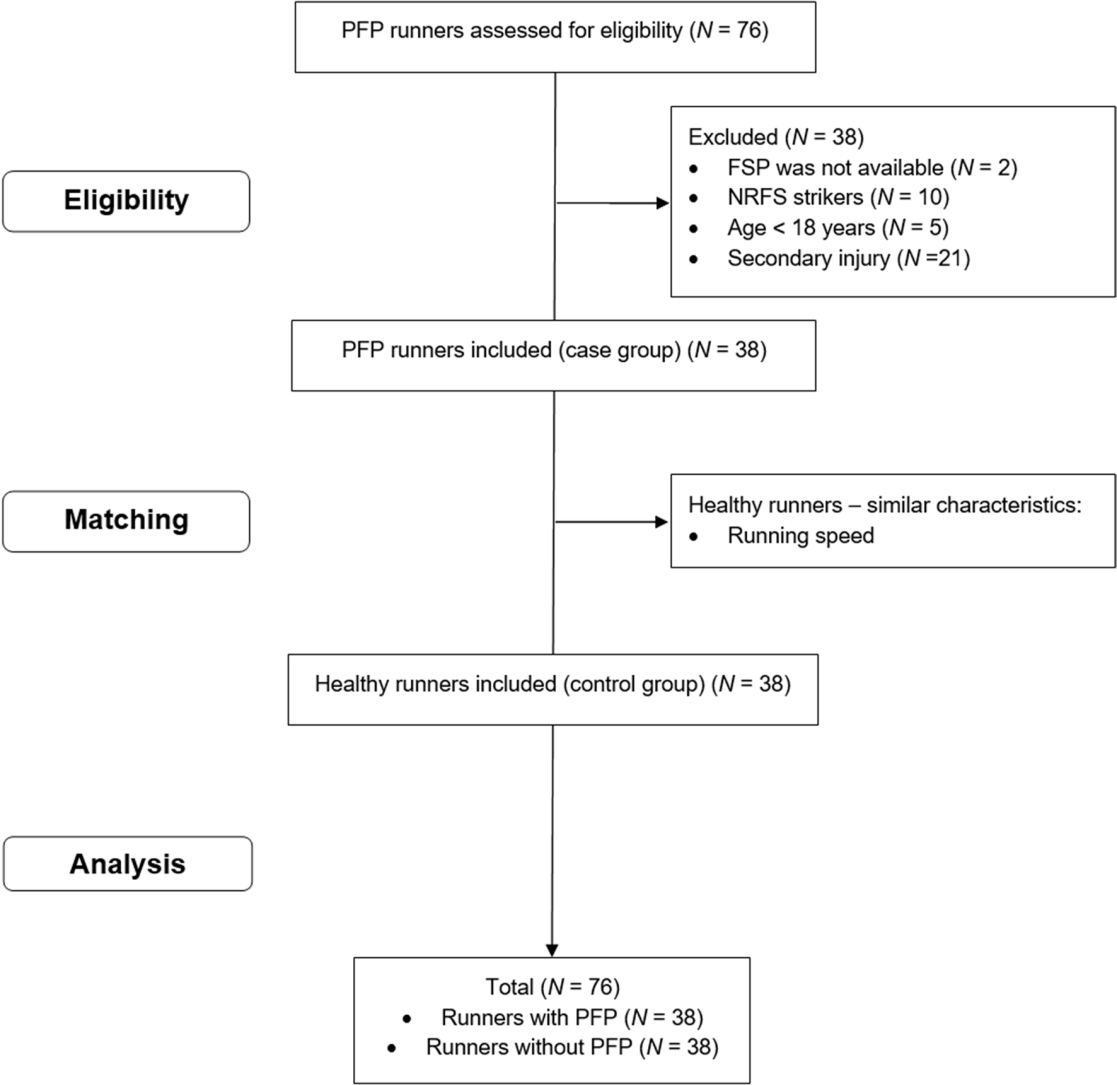
Study flowchart. FSP = foot strike pattern; NRFS = non-rearfoot strike pattern; PFP = patellofemoral pain

Data were collected from September 2012 to July 2022. Review of clinical charts from August 2022 to October 2022 identified 38 runners with PFP who met the inclusion criteria (Fig. 1). Therefore, 38 healthy controls were matched according to self-selected running speed. No between-group difference was found for this aspect (PFP = 2.54 (0.2) m/s x CON = 2.55 (0.1) m/s, *P* = 0.660). Patients reported months experiencing symptoms of PFP, with a range from 0.5 months to 163 months reported. Descriptive data for group characteristics are presented in Table 1.

**Table 1:** Personal and biomechanical data for those with and without PFP (n = 76)

Variables	Total (N = 76)	PFP (N = 38)	CON (N = 38)
Sex. %			
Male Female	42 (55.3) 34 (44.7)	24 (63.2) 14 (36.8)	18 (47.4) 20 (52.6)
Age, y	33 (16)	34 (15)	31 (19)
Weight, kg	65.6 (15.7)	65.1 (14)	67.5 (17.5)
Height, cm	172.1 (18.4)	173.2 (8.4)	169.6 (21.4)
BMI, kg/m^2^	22.3 (2.6)	22.3 (2.3)	22.2 (2.3)
VALR, BW/s	60.3 (25.5)	67.8 (21.3)	55.7 (23.1)
VILR, BW/s	69.4 (30.9)	77.2 (22.9)	65.8 (25.8)
BGRFI, BW*s	0.014 (0.002)	0.014 (0.002)	0.015 (0.002)
Contact Time, s	0.26 (0.02)	0.27 (0.02)	0.26 (0.02)
Step rate, steps/min	165.2 (9.6)	167.0 (8.9)	165.6 (10.4)

^a^BGRFI = braking ground reaction force impulse; BMI = body mass index; CON = controls; PFP = patellofemoral pain; VALR = vertical average loading rate; VILR = vertical instantaneous loading rate

^b^Continuous variables = mean (SD) or median (IQR)

^c^Categorical variables = n (%)

**Fig. 2:**
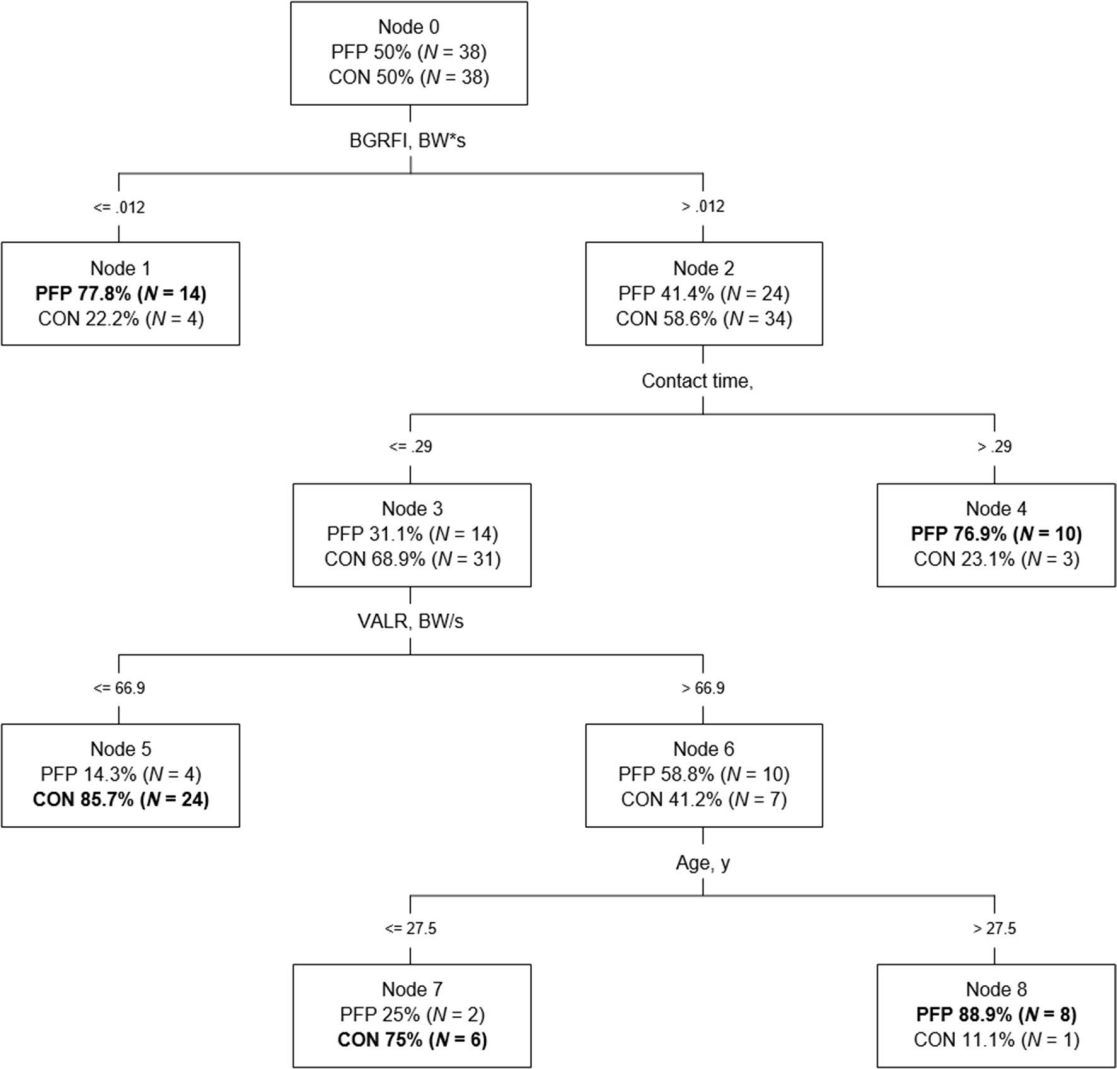
Classification and regression tree model for Patellofemoral Pain. BGRFI braking ground reaction force impulse; CI = confidence interval; CON = controls; PFP = presence of patellofemoral pain; PR = prevalence ratio; VALR = vertical average loading rate. Bold represents the largest proportion of subjects classified as PFP or CON at the terminal node

The correlation matrix identified interactions between VALR and VILR (r = 0.99/ *P* < 0.001). To avoid multicollinearity, VILR was removed from the CART analysis. Figure 2 illustrates the final model of the 4-level decision tree, including 8 nodes and 5 terminal subgroups. Terminal nodes 1, 4, and 8 classified runners with PFP while nodes 5 and 7 classified healthy runners. The CART analyses showed that the variables of BGRFI, contact time, VALR, and age identified runners with PFP. The CART model correctly classified 32 (84.2%) runners with PFP, and 30 (78.9%) of the healthy runners. Correct classification was achieved in most runners (81.6%), and the area under the ROC curve (accuracy) was 0.83 (95% CI 0.74–0.93; SE, 0.04; *P* < 0.001).

Node 1 (low BGRFI), node 4 (high BGRFI and low contact time), and node 8 (high BGRFI, low contact time, high VALR and older age) accurately classified participants with PFP. Node 5 (high BGRFI, low contact time, low VALR) and node 7 (high BGRFI, low contact time, high VALR, and young age) accurately classified participants without PFP. Specific cut-off values are presented in Fig. 2. Statistically significant differences in the proportion of runners with and without PFP were found in nodes 1, 4, 5, and 8 (*P* < 0.05) (Table 2).

**Table 2:** Prevalence Ratios and 95% Confidence Intervals for the profiles identified through the model (n = 76)

	PR (95% CI)
**Profiles for runners with PFP**	
**Node 1** Braking Ground Reaction Force Impulse < = .012 BW*s	4.95 (1.45–16.93)*
**Node 4** Braking Ground Reaction Force Impulse > .012 BW*s Contact time > .29 s	4.16 (1.04–16.60)*
**Node 8** Braking Ground Reaction Force Impulse > .012 BW*s Contact time < = .29 s Average Vertical Loading Rate > 66.9 BW/s Age > 27.5 y	9.86 (1.16–83.34)*
**Profiles for healthy runners**	
**Node 5** Braking Ground Reaction Force Impulse > .012 BW*s Contact time < = .29 s Average Vertical Loading Rate < = 66.9 BW/s	0.06 (0.02–0.23)*
**Node 7** Braking Ground Reaction Force Impulse > .012 BW*s Contact time < = .29 s Average Vertical Loading Rate > 66.9 BW/s Age < = 27.5 y	0.29 (0.05–1.57)

^a^CI = confidence interval. PFP = patellofemoral pain. PR = prevalence ratio

^*^P < .05, proportion of participants with and without PFP

## Discussion

The purpose of this study is to characterize differences in runners with PFP compared to uninjured runners using CART analysis. We hypothesized runners with PFP would exhibit different patterns of interactions among predictor variables compared to healthy controls. As expected, interactions among BGRFI, contact time, VALR, and age were captured through CART analysis. The interactions of different variables and combination of factors associated with PFP illustrates the challenges in isolating single risk factors for this condition.

BGRFI was the first variable to enter the CART model. The observed lower BGRFI in runners with PFP is consistent with a prior systematic review on biomechanical risk factors for RRIs that showed moderate evidence for a reduced BGRFI in runners with PFP [19]. It could be hypothesized that a reduced BGRFI could be related to a reduced knee extensor moment. Theoretically, this node may indicate a subgroup of participants that adopt a quadriceps avoidance running pattern. In one prior study, lower peak resultant patellofemoral joint reaction forces (PFJRFs) during running were found in females with PFP. The reductions in PFJRFs were seen in parallel with a reduction in knee extensor moment and were explained as a compensatory measure [37]. Therefore, rather than identify a risk, this node may reflect those runners who are trying to compensate for their symptoms.

We also noted that runners with PFP may have *higher* BGRFI through an interaction with greater contact time. Moderate evidence for higher contact time was reported in runners with PFP [19]. Previous studies have shown that a reduced step rate was related to higher BGRFI and greater contact time [38]. Theoretically, this node may indicate a subgroup of participants with an overstride posture. This pattern may lead to greater knee flexion excursion during stance and increase negative knee work. An association between components of an overstride posture with peak knee flexion, quadriceps peak force, knee extensor moment, peak patellofemoral force and negative knee work was reported previously [38, 39]. The association between an overstride posture and PFP should be studied prospectively.

Runners who had increased BGRFI, and reduced contact time had an observed interaction in the CART model when accounting for elevated VALR and older age. Higher load rates were found in runners with PFP when compared with healthy controls [15]. It is intuitive that higher load rates may result in greater demands to the PF joint. Older runners present less contact time, leg stiffness, and knee excursion [40]. Theoretically, this pattern together with age-tissue changes such as reduction of skeletal muscle mass and muscle strength could potentially compromise the ability of the PF joint to dissipate energy. Previously, runners with PFP presented higher vertical stiffness [15] and reductions in relative skeletal muscle starting in the third decade were reported [41].

It was interesting to note that sex was not a significant factor in PFP given that it has been reported and is commonly stated that females are twice as likely to have PFP than their male counterparts. This may have been a function of the sex distribution of this study, whereby 24 of the PFP group were males and only 14 were female [24, 25]. This was a cross-sectional study with a convenience sample of runners with PFP. This sample reflects only the patients who sought care in the clinic. Our study may lack the power to detect differences regarding sex and that a prospective study may promote a different view regarding this aspect.

To our knowledge, this study is the first to assess the interactions between ground reaction forces, spatiotemporal, anthropometric, and demographic data through CART to characterize factors associated with PFP in runners. The model may have clinical implications when considering what biomechanical factors to include when evaluating the biomechanics of a runner with PFP. Results from nodes 1 and 8 may indicate that appropriate rehabilitation may be needed to improve the capacity of the knee joint to take further load during running. Results from node 4 may indicate that changes in running biomechanics may be required to impose less demand on the knee joint during this activity. Clinicians should focus on the identification of which interactions may represent a cause and which ones may represent a consequence. The goal is to recognize the pattern presented in the runner with PFP and choose the more suitable approach to help with their symptoms.

There are limitations to this study. The results must be interpreted with caution due to the very wide confidence intervals presented in the terminal nodes. Causal relationships cannot be inferred due to the design of the analysis. Also, a larger sample could provide different insight regarding the profiles of runners with PFP. The runners analyzed were limited to rearfoot strike runners, therefore it is unclear whether similar results would be obtained in non-rearfoot strike runners. Some evidence suggest that hip kinematics may be associated with PFP [19]. Unfortunately, kinematic data were not available for the PFP participants. Prior work has suggested training factors are strongly associated with RRIs [5], however, these were not available in all runners. While the analysis did consider BMI, other aspects of body composition were not measured. Higher body fat and lower skeletal muscle mass have been observed in women with PFP and these factors are associated with pressure hyperalgesia [42, 43]. PFP is influenced by a variety of biopsychosocial factors such as catastrophizing and pain-related fear [44]; the present study considered primarily the biomechanical factors. Future studies should include some of these aspects along with the variables presented in our paper.

## Conclusions

In conclusion, BGRFI, contact time, VALR, and age were the factors which best identified runners with current PFP. The model with these aspects correctly identified 84.2% of runners with PFP, and 78.9% of runners without PFP. The total correct classification was 81.6%. The area under the ROC curve was 0.83 indicating that our results were not due to chance. The profile that best classified participants with PFP was BGRFI higher than 0.012 BW*s, contact time lower than 0.29 s, VALR higher than 66.9 BW/s, and being older than 27.5 years of age. Interesting, step rate, sex, and BMI did not enter in the CART model. More research is needed to determine if these are causative or compensatory factors. Our results may serve as a basis for future prospective studies with the goal of identifying risk and protective profiles for PFP in runners.

## Data Availability

The datasets analyzed during the current study are available from the corresponding author on reasonable request.

## Abbreviations

BGRFI: Braking ground reaction force impulse
BMI: Body mass index
BW: Body weight
CART: Classification and regression tree
CI: Confidence interval
CON: Control group
FSP: Foot strike pattern
GRF: Ground reaction force
IQR: Interquartile range
NRFS: Non-rearfoot strike pattern
PFJRF: Patellofemoral joint reaction force
PFP: Patellofemoral pain
POI: Point of interest
PR: Prevalence ratio
ROC: Receiver operating characteristic
RRI: Running-related injury
SD: Standard deviation
SPSS: Statistical package for social sciences
STROBE: Strengthening the reporting of observational studies in epidemiology
VALR: Vertical average loading rate
VILR: Vertical instantaneous loading rate

## References

[1] Hespanhol Junior LC, Pillay JD, van Mechelen W, Verhagen E (2015). Meta-analyses of the effects of habitual running on indices of health in physically inactive adults. Sports Med.

[2] Van Gent RN, Siem D, van Middelkoop M, van Os AG, Bierma-Zeinstra SMA, Koes BW (2007). Incidence and determinants of lower extremity running injuries in long distance runners: a systematic review. Br J Sports Med.

[3] Kemler E, Blokland D, Backx F, Huisstede B (2018). Differences in injury risk and characteristics of injuries between novice and experienced runners over a 4-year period. Physician Sportsmed.

[4] Videbæk S, Bueno AM, Nielsen RO, Rasmussen S (2015). Incidence of running-related injuries per 1000 h of running in different types of runners: a systematic review and meta-analysis. Sports Med.

[5] Van der Worp MP, ten Haaf DSM, van Cingel R, de Wijer A, Nijhuis-van der Sanden MWG, Staal JB (2015). Injuries in runners; a systematic review on risk factors and sex differences. PLoS ONE.

[6] Crossley KM, Stefanik JJ, Selfe J, Collins NJ, Davis IS, Powers CM (2016). Patellofemoral pain consensus statement from the 4th International Patellofemoral Pain Research Retreat, Manchester. Part 1: Terminology, definitions, clinical examination, natural history, patellofemoral osteoarthritis and patient-reported outcome measures. Br J Sports Med.

[7] Smith BE, Selfe J, Thacker D, Hendrick P, Bateman M, Moffatt F (2018). Incidence and prevalence of patellofemoral pain: a systematic review and meta-analysis. PLoS ONE.

[8] Lopes AD, Hespanhol LC, Yeung SS, Costa LOP (2012). What are the main running-related musculoskeletal injuries?. Sports Med.

[9] Collins NJ, Bierma-Zeinstra SMA, Crossley KM, van Linschoten RL, Vicenzino B, van Middelkoop M (2012). Prognostic factors for patellofemoral pain: a multicentre observational analysis. Br J Sports Med.

[10] Lankhorst NE, van Middelkoop M, Crossley KM, Bierma-Zeinstra SMA, Oei EHG, Vicenzino B (2015). Factors that predict a poor outcome 5–8 years after the diagnosis of patellofemoral pain: a multicentre observational analysis. Br J Sports Med.

[11] Lankhorst NE, Bierma-Zeinstra SMA, van Middelkoop M (2012). Factors associated with patellofemoral pain syndrome: a systematic review. Br J Sports Med.

[12] Lankhorst NE, Bierma-Zeinstra SMA, van Middelkoop M (2012). Risk factors for patellofemoral pain syndrome: a systematic review. J Orthop Sports Phys Ther.

[13] Neal BS, Lack SD, Lankhorst NE, Raye A, Morrissey D, van Middelkoop M (2018). Risk factors for patellofemoral pain: a systematic review and meta-analysis. Br J Sports Med.

[14] Davis IS, Bowser BJ, Mullineaux DR (2016). Greater vertical impact loading in female runners with medically diagnosed injuries: a prospective investigation. Br J Sports Med.

[15] Johnson CD, Tenforde AS, Outerleys J, Reilly J, Davis IS (2020). Impact-related ground reaction forces are more strongly associated with some running injuries than others. Am J Sports Med.

[16] Messier SP, Davis SE, Curl WW, Lowery RB, Pack RJ (1991). Etiologic factors associated with patellofemoral pain in runners. Med Sci Sports Exerc.

[17] Esculier JF, Roy JS, Bouyer LJ (2015). Lower limb control and strength in runners with and without patellofemoral pain syndrome. Gait Posture.

[18] Duffey MJ, Martin DF, Cannon DW, Craven T, Messier SP (2000). Etiologic factors associated with anterior knee pain in distance runners. Med Sci Sports Exerc.

[19] Willwacher S, Kurz M, Robbin J, Thelen M, Hamill J, Kelly L, Mai P (2022). Running-related biomechanical risk factors for overuse injuries in distance runners: a systematic review considering injury specificity and the potentials for future research. Sports Med.

[20] Luedke LE, Heiderscheit BC, Williams DS, Rauh MJ (2016). Influence of step rate on shin injury and anterior knee pain in high school runners. Med Sci Sports Exerc.

[21] Esculier J-F, Bouyer LJ, Dubois B, Fremont P, Moore L, McFadyen B (2017). Is combining gait retraining or an exercise programme with education better than education alone in treating runners with patellofemoral pain? A randomised clinical trial. Br J Sports Med.

[22] Dos Santos AF, Nakagawa TH, Lessi GC, Luz BC, Matsuo HTM, Nakashima GY (2019). Effects of three gait retraining techniques in runners with patellofemoral pain. Phys Ther Sport.

[23] Bramah C, Preece SJ, Gill N, Herrington L (2019). A 10% Increase in step rate improves running kinematics and clinical outcomes in runners with patellofemoral pain at 4 weeks and 3 months. Am J Sports Med.

[24] Taunton JE, Ryan MB, Clement DB, McKenzie DC, Lloyd-Smith DR, Zumbo BD (2002). A retrospective case-control analysis of 2002 running injuries. Br J Sports Med.

[25] Boling M, Padua D, Marshall S, Guskiewicz K, Pyne S, Beutler A (2010). Gender differences in the incidence and prevalence of patellofemoral pain syndrome. Scand J Med Sci Sports.

[26] Hart HF, Barton CJ, Khan KM, Riel H, Crossley KM (2016). Is body mass index associated with patellofemoral pain and patellofemoral osteoarthritis? A systematic review and meta-regression and analysis. Br J Sports Med.

[27] Bittencourt NFN, Meeuwisse WH, Mendonça LD, Nettel-Aguirre A, Ocarino JM, Fonseca ST (2016). Complex systems approach for sports injuries: moving from risk factor identification to injury pattern recognition—narrative review and new concept. Br J Sports Med.

[28] Philippe P, Mansi O (1998). Nonlinearity in the epidemiology of complex health and disease processes. Theor Med Bioeth.

[29] Lemon SC, Roy J, Clark MA, Friedmann PD, Rakowski W (2003). Classification and regression tree analysis in public health: methodological review and comparison with logistic regression. Ann Behav Med.

[30] Ferreira VMLM, Oliveira RR, Nazareno TS, Freitas LV, Mendonça LD (2020). Interaction of foot and hip factors identifies Achilles tendinopathy occurrence in recreational runners. Phys Ther Sport.

[31] Fältström A, Kvist J, Bittencourt NFN, Mendonça LD, Hägglund M (2021). Clinical risk profile for a second anterior cruciate ligament injury in female soccer players after anterior cruciate ligament reconstruction. Am J Sports Med.

[32] Futrell EE, Jamison ST, Tenforde AS, Davis IS (2018). Relationships between habitual cadence, footstrike, and vertical load rates in runners. Med Sci Sports Exerc.

[33] Hollander K, Johnson CD, Outerleys J, Davis IS (2020). Multifactorial determinants of running injury locations in 550 injured recreational runners. Med Sci Sports Exerc.

[34] Tenforde AS, Ruder MC, Jamison ST, Singh PP, Davis IS (2018). Is symmetry of loading improved for injured runners during novice barefoot running?. Gait Posture.

[35] Breiman L, Friedman JH, Olshen RA, Stone CJ. Regression Trees. In: Classification and regression trees. Routledge; 2017.

[36] Chester R, Khondoker M, Shepstone L, Lewis JS, Jerosch-Herold C (2019). Self-efficacy and risk of persistent shoulder pain: results of a classification and regression tree (CART) analysis. Br J Sports Med.

[37] Chen YJ, Powers CM (2014). Comparison of three-dimensional patellofemoral joint reaction forces in persons with and without patellofemoral pain. J Appl Biomech.

[38] Anderson LM, Martin JF, Barton CJ, Bonanno DR (2022). What is the effect of changing running step rate on injury, performance and biomechanics? A systematic review and meta-analysis. Sports Med Open.

[39] Wille CM, Lenhart RL, Wang S, Thelen DG, Heiderscheit BC (2014). Ability of sagittal kinematic variables to estimate ground reaction forces and joint kinetics in running. J Orthop Sports Phys Ther.

[40] Willy RW, Paquette MR (2019). The physiology and biomechanics of the master runner. Sports Med Arthrosc Rev.

[41] Janssen I, Heymsfield SB, Wang ZM, Ross R (1985). Skeletal muscle mass and distribution in 468 men and women aged 18–88 yr. J Appl Physiol.

[42] Ferreira AS, Mentiplay BF, Taborda B, Pazzinatto MF, de Azevedo FM, De Oliveira SD (2021). Exploring overweight and obesity beyond body mass index: a body composition analysis in people with and without patellofemoral pain. J Sport Health Sci.

[43] Ferreira AS, Lack S, Taborda B, Pazzinatto MF, de Azevedo FM, De Oliveira SD (2022). Body fat and skeletal muscle mass, but not body mass index, are associated with pressure hyperalgesia in young adults with patellofemoral pain. Braz J Phys Ther.

[44] Maclachlan LR, Collins NJ, Matthews MLG, Hodges PW, Vicenzino B (2017). The psychological features of patellofemoral pain: a systematic review. Br J Sports Med.

